# Drought of early time in growing season decreases community aboveground biomass, but increases belowground biomass in a desert steppe

**DOI:** 10.1186/s12862-021-01842-5

**Published:** 2021-06-01

**Authors:** Xiangyun Li, Xiaoan Zuo, Ping Yue, Xueyong Zhao, Ya Hu, Xinxin Guo, Aixia Guo, Chong Xu, Qiang Yu

**Affiliations:** 1 Urat Desert-Grassland Research Station, Northwest Institute of Eco-Environment and Resources, Chinese Academy of Science, Lanzhou, 730000 China; 2 University of Chinese Academy of Sciences, Beijing, 100049 China; 3 Naiman Desertification Research Station, Northwest Institute of Eco-Environment and Resources, Chinese Academy of Science, Lanzhou, 730000 China; 4 Key Laboratory of Stress Physiology and Ecology in Cold and Arid Region, Gansu Province, Lanzhou, 730000 China; 5 National Hulunber Grassland Ecosystem Observation and Research Station, Institute of Agricultural Resources and Regional Planning, Chinese Academy of Agricultural Sciences, Beijing, 10008 China

**Keywords:** Precipitation changes, Species diversity, Plant traits, Plant biomass

## Abstract

**Background:**

Increasing drought induced by global climate changes is altering the structure and function of grassland ecosystems. However, there is a lack of understanding of how drought affects the trade-off of above- and belowground biomass in desert steppe. We conducted a four-year (2015–2018) drought experiment to examine the responses of community above-and belowground biomass (AGB and BGB) to manipulated drought and natural drought in the early period of growing season (from March to June) in a desert steppe. We compared the associations of drought with species diversity (species richness and density), community-weighted means (CWM) of five traits, and soil factors (soil Water, soil carbon content, and soil nitrogen content) for grass communities. Meanwhile, we used the structural equation modeling (SEM) to elucidate whether drought affects AGB and BGB by altering species diversity, functional traits, or soil factors.

**Results:**

We found that manipulated drought affected soil water content, but not on soil carbon and nitrogen content. Experimental drought reduced the species richness, and species modified the CWM of traits to cope with a natural drought of an early time in the growing season. We also found that the experimental and natural drought decreased AGB, while natural drought increased BGB. AGB was positively correlated with species richness, density, CWM of plant height, and soil water. BGB was negatively correlated with CWM of plant height, CWM of leaf dry matter content, and soil nitrogen content, while was positively correlated with CWM of specific leaf area, CWM of leaf nitrogen content, soil water, and soil carbon content. The SEM results indicated that the experimental and natural drought indirectly decreased AGB by reducing species richness and plant height, while natural drought and soil nitrogen content directly affected BGB.

**Conclusions:**

These results suggest that species richness and functional traits can modulate the effects of drought on AGB, however natural drought and soil nitrogen determine BGB. Our findings demonstrate that the long-term observation and experiment are necessary to understand the underlying mechanism of the allocation and trade-off of community above-and belowground biomass.

**Supplementary Information:**

The online version contains supplementary material available at 10.1186/s12862-021-01842-5.

## Background

Grasslands occupy 30% of Earth’s terrestrial area, while also responding sensitively to climate changes [1, 2]. Global climatic changes are expected to increase the risk of extreme drought events [3, 4]. Drought has pervasive impacts on ecosystem structure and function, especially in water-limited grasslands [5, 6]. Several previous studies have observed that the plant community biomass tends to increase, decrease, or remain stable under drought [7–10]. In addition, drought is one of the main drivers that determine species diversity and leaf morphology [11, 12]. Previous studies have shown that species diversity and functional traits were thought to play a vital role in affecting ecosystem function [13, 14]. These responses of plant to water restriction may relate to the type of drought experienced by an ecosystem [15]. Grasslands are expected to experience a decrease in long-term precipitation, and an increase in the frequency of short-term intense droughts [16, 17]. Meanwhile, some studies have suggested that changes in the timing of drought events can alter plant productivity regardless of precipitation amount [18, 19]. However, we have a very limited understanding of how timing and type of drought affect species diversity, functional traits, and plant biomass and their relationships in desert steppe.

The impact of climate change on biodiversity is greater than any other factor [20]. The control of species diversity including species richness and abundance is received the most focus [20, 21]. The relationship between ecosystem productivity and species diversity has been debated for decades [22]. In general, higher species diversity supports higher plant productivity but remains variation in other geographic regions [20, 23]. Globally, regions with a climate that is either cold or arid support few species than regions where the climates are both warm and wet [11]. Most species diversity-biomass relationship studies have focused on aboveground biomass instead of belowground biomass [13]. In a few studies on the relationship between belowground biomass and species diversity, it was found that there was a positive or uncorrelated relationship between them, due to the selection of diversity indexes and the research sites [24–27]. Plant biomass is important for ecosystem functions and services [28]. Therefore, examining the drought-induced relationship between biodiversity and biomass can provide support for further understanding of ecosystem management.

Functional traits are measurable characteristics of plants after long-term response and adaptation to the external environment [29, 30]. According to the dominance/mass ratio hypothesis, the functional trait of dominant species can directly affect ecosystem functions [31, 32]. Some traits at a community-level are the predictors of plant community responses to precipitation changes [33, 34]. Shifts in precipitation patterns can lead to changes in traits and species abundance, thereby shaping plant distributions or compositions [12]. The key plant traits, such as plant height, specific leaf area (SLA), leaf dry matter content (LDMC), leaf carbon content (LCC), and leaf nitrogen content (LNC), reflect plant strategies for coping with changing climate conditions [35]. For example, drought or drought in the growing season causes a decrease in plant height and an increase in SLA and LDMC [36]. According to the optimal allocation theory, plants preferentially develop the organs that can obtain the most limited growth resources [37]. Thus, the plant functional traits as potential covariates may lead to the trade-off of biomass under drought [14, 38]. However, the effects of drought-induced changes in plant functional traits on aboveground and belowground biomass remain poorly known [14]. Arid and semi-arid grassland, which is sensitive to precipitation changes, plays an important role in maintaining regional ecosystem function and socioeconomic development [37]. Therefore, understanding the relationship between drought-induced functional traits and biomass is important to understand the consequences of precipitation pattern changes in this region.

Precipitation manipulation experiment is a direct way to study shifts in community compositions and ecosystem functions following short-term precipitation change [39, 40]. Over the two decades, the studies on experimentally reducing precipitation have greatly increased to investigate how increased aridity might influence the ecosystems [41, 42]. However, community responses to extreme drought vary geographically [43, 44]. The semiarid grassland region of northern China is desirable for investigating the effects of extreme drought on the structure and function of grassland ecosystems, and the predicted effects can guide semiarid grassland to cope with future climate change. Here, we conducted a four-year experiment that imposed extreme drought, including two types: (1) a 66% reduction of rainfall from May to August (-66%) and (2) a 100% reduction of rainfall from June to July (-60 Days). This allowed us to examine the changes in the desert steppe under a manipulative drought experiment. We asked the following questions: (1) Whether the responses of species diversity, community-level trait, and soil property respond to the two types of experimental drought in desert steppe are different? (2) How do changes the above- and belowground biomass in the four consecutive years under different treatments? (3) How does extreme drought modulate the relationships between species diversity, community-level traits, and above- and belowground biomass?

## Results

The annual precipitation gradually decreased from 2015 to 2017 and increased in 2018 due to increased precipitation in July and August (Additional file 1: Figure S1). Interestingly, only the precipitation in the early growing season (March to June) was correlated with the above- and belowground biomass (Additional file 1: Table S1). The precipitation of early time in the growing season (March to June) from 2015 to 2018 was 56.2 mm, 77.9 mm, 28.8 mm, and 21.4 mm, respectively (Additional file 1: Figure S1), which described as a natural interannual drought phenomenon. As a result, the strong correlation showed that drought of early time in growing season played an important role for the plant biomass.

**Fig. 1:**
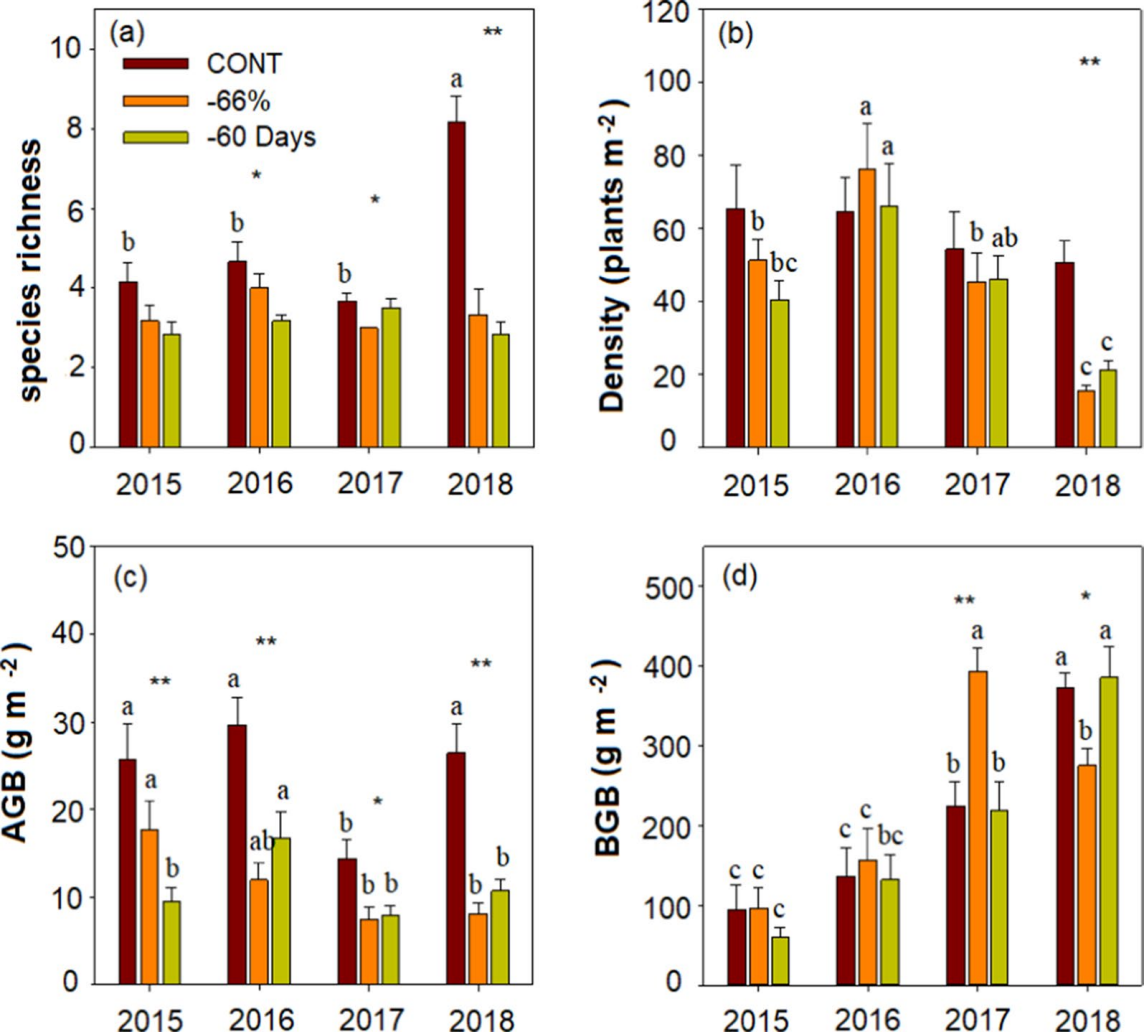
Effects of extreme drought (CONT, control; − 66%, reduce 66% in rainfall from May to August; − 60 Days, reduce 100% in rainfall from June to July) on plant community characteristics of desert steppe during the treatment years (2015–2018). *AGB* aboveground plant biomass, *BGB* belowground root biomass. Variables are shown as mean ± SE (n = 6). Different lowercase letters indicate significant differences between years for the same treatments in p < 0.05. Statistical significance of drought effect in each year is depicted as **p < 0.0 1 and *p < 0.05

There were no differences in above- and belowground biomass, species diversity, CWM of traits and soil carbon and nitrogen content between -66% and -60 Days (Additional file 1: Table S2), so we combined the two treatments to represent experimental drought in the description below. The species richness and AGB were significantly affected by drought, year and their interaction (*p* < 0.05, Additional file 1: Table S3, Fig. 1a, c). Specifically, manipulated drought (-66% and -60 Days) significantly reduced AGB and species richness excluded species richness in 2015(Additional file 1: Table S2, Fig. 1a, c). The density was positively corrected with drought and year, and the BGB was significantly affected by year and the interaction of drought and year (*p* < 0.05, Additional file 1: Table S3, Fig. 1b, d). Surprisedly, BGB was increased with years, regardless of manipulated drought (Additional file 1: Table S2, Fig. 1d).

**Fig. 2:**
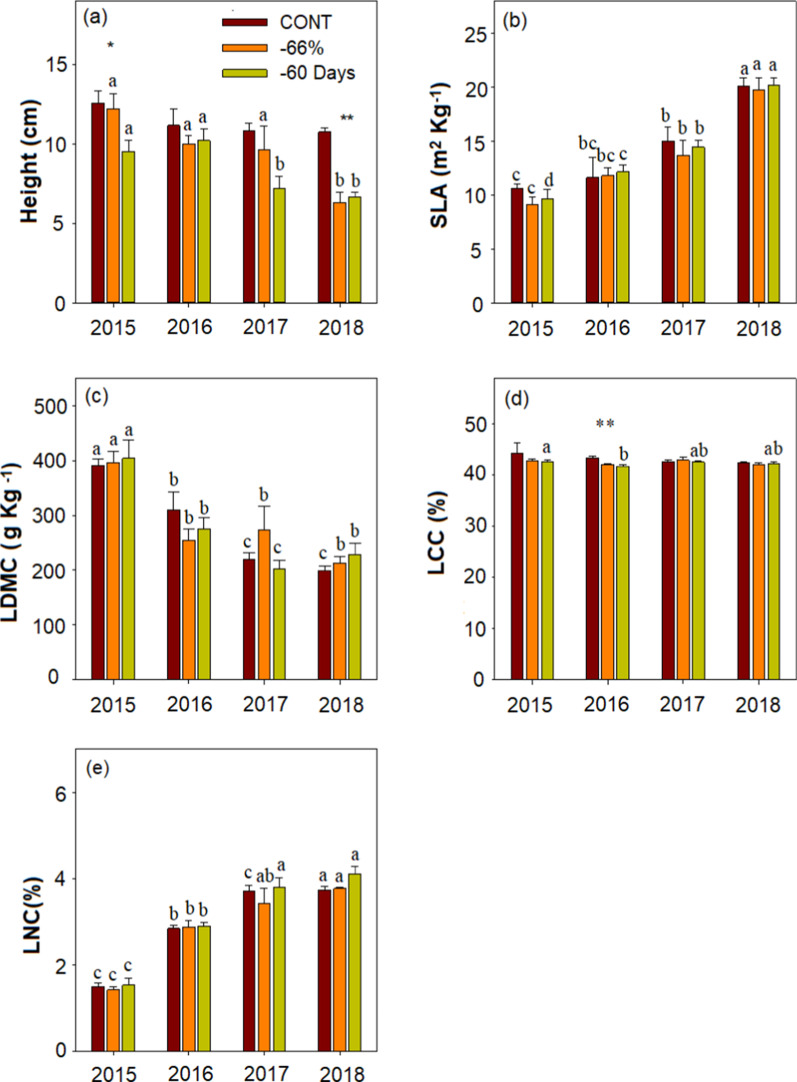
Effects of extreme drought (CONT, control; − 66%, reduce 66% in rainfall from May to August; − 60 Days, reduce 100% in rainfall from June to July) on plant community trait during the treatment years (2015–2018). *SLA* specific leaf area, *LDMC* leaf dry matter content, *LCC* leaf carbon content, *LNC* leaf nitrogen content. Variables are shown as mean ± SE (n = 6). Different lowercase letters indicate significant differences between years for the same treatments in p < 0.05. Statistical significance of drought effect in each year is depicted as **p < 0.0 1 and *p < 0.05

The CWM of height in desert steppe was significantly affected by drought, year and their interaction (Additional file 1: Table S3, Fig. 2a). The CWM of height in 2018 was significantly lower than that in 2015–2016 under manipulated drought (− 66% and − 60 Days) (p < 0.05; Fig. 2a). However, CWM of SLA, LDMC, and LNC were largely varied across years (Additional file 1: Table S3, Fig. 2b, c, e). CWM of SLA, and LNC increased following the year and reached their maximum in 2018 (Fig. 2b, e). In contrast, CWM of LDMC was decreased following the year and reached their minimum in 2018 (Fig. 2c). CWM of LCC had significant differences only under the manipulated drought of 60 days in 2015–2016 (Fig. 2d).

**Fig. 3:**
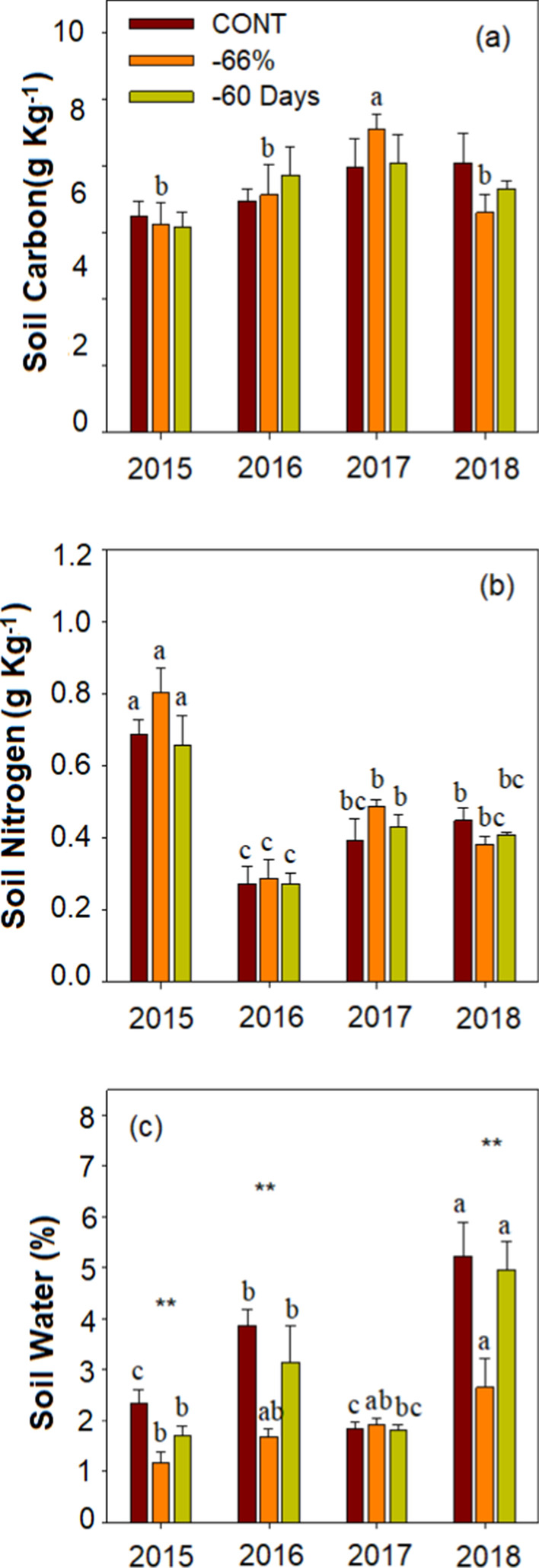
Effects of extreme drought (CONT, control; − 66%, reduce 66% in rainfall from May to August; − 60 Days, reduce 100% in rainfall from June to July) on soil characteristics during the treatment years (2015–2018). Soil Carbon, 0–20 cm soil carbon content; Soil Nitrogen, 0–20 cm soil nitrogen content; Soil Water, 0–20 cm soil water content. Variables are shown as mean ± SE (n = 6). Different lowercase letters indicate significant differences between years for the same treatments in p < 0.05. Statistical significance of drought effect in each year is depicted as ** p < 0.0 1 and * p < 0.05

Drought, year and their interaction had a significant influence on soil water content (p < 0.05, Additional file 1: Table S3, Fig. 3c). There were significant differences in soil water content between 2015 and 2016 and 2018 under different drought treatments (p < 0.05; Fig. 3c). The soil water content in 2015 and 2017 was significantly lower than that in 2016 and 2018 under CONT, while significantly higher in 2018 than that in 2015 under -66% and -60 Days drought treatment (p < 0.05; Fig. 3c). The soil carbon content and soil nitrogen content are only affected by years (p < 0.01, Additional file 1: Table S3, Fig. 3a, b). Under − 66% treatment, the soil carbon content in 2017 was significantly higher than that in 2015–2016 and 2018, and soil nitrogen content in 2015 was significantly higher than that in 2016–2018 (p < 0.05; Fig. 3a, b).

**Fig. 4:**
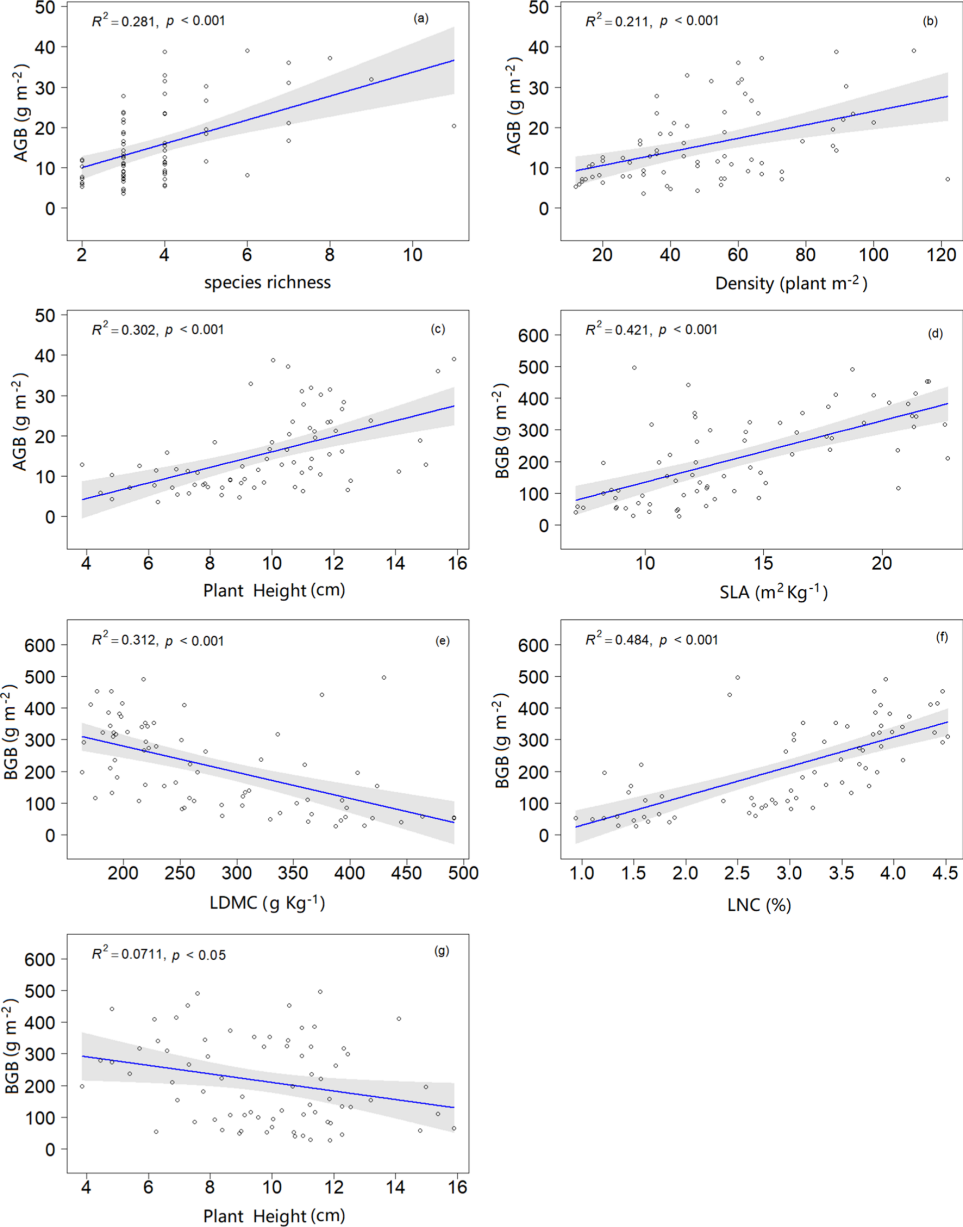
Relationships of community above-and belowground biomass with species diversity and community-weighted functional traits across four years in the desert steppe. Only significant (p ≤ 0.05) relationships were shown. *SLA* specific leaf area, *LDMC* leaf dry matter content, *LNC* leaf nitrogen content

**Fig. 5:**
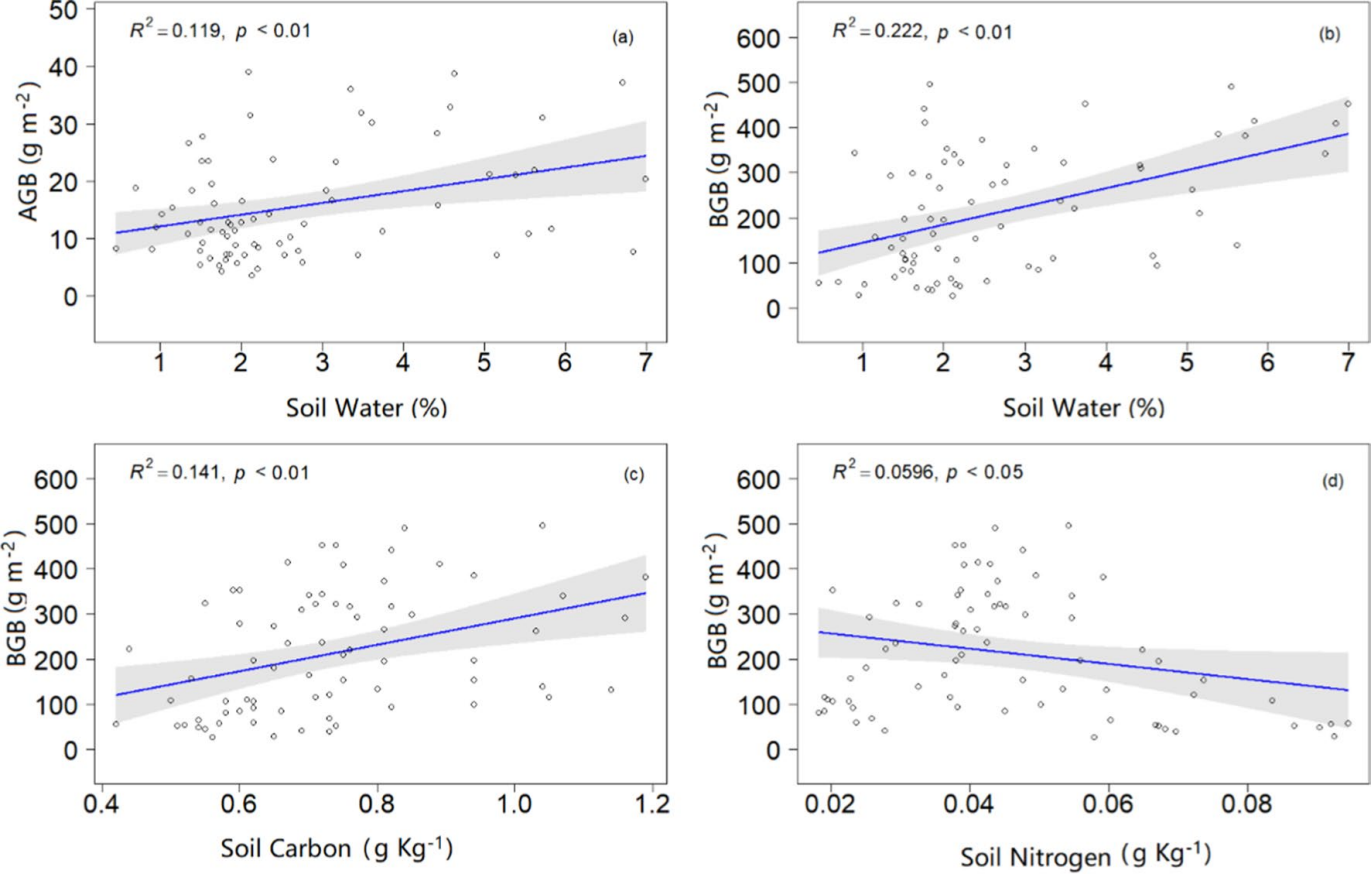
Relationships of community above-and belowground biomass with soil factors across four years in the desert steppe. Only significant (p ≤ 0.05) relationships were shown

Across the four years, AGB was positive correlated with species diversity (species richness and density) (p < 0.001; Fig. 4a, b), CWM of plant height (p < 0.001; Fig. 4c) and soil water (p < 0.01; Fig. 5a). BGB was positive correlated with CWM of SLA (p < 0.001; Fig. 4d), LNC (p < 0.001; Fig. 4f), soil water (p < 0.01; Fig. 5b) and soil carbon (p < 0.01; Fig. 5c). However, we found significant negative relationships between BGB and CWM of LDMC (p < 0.001; Fig. 4e), plant height (p < 0.05; Fig. 4g) and soil nitrogen (p < 0.05; Fig. 5d).

**Fig. 6:**
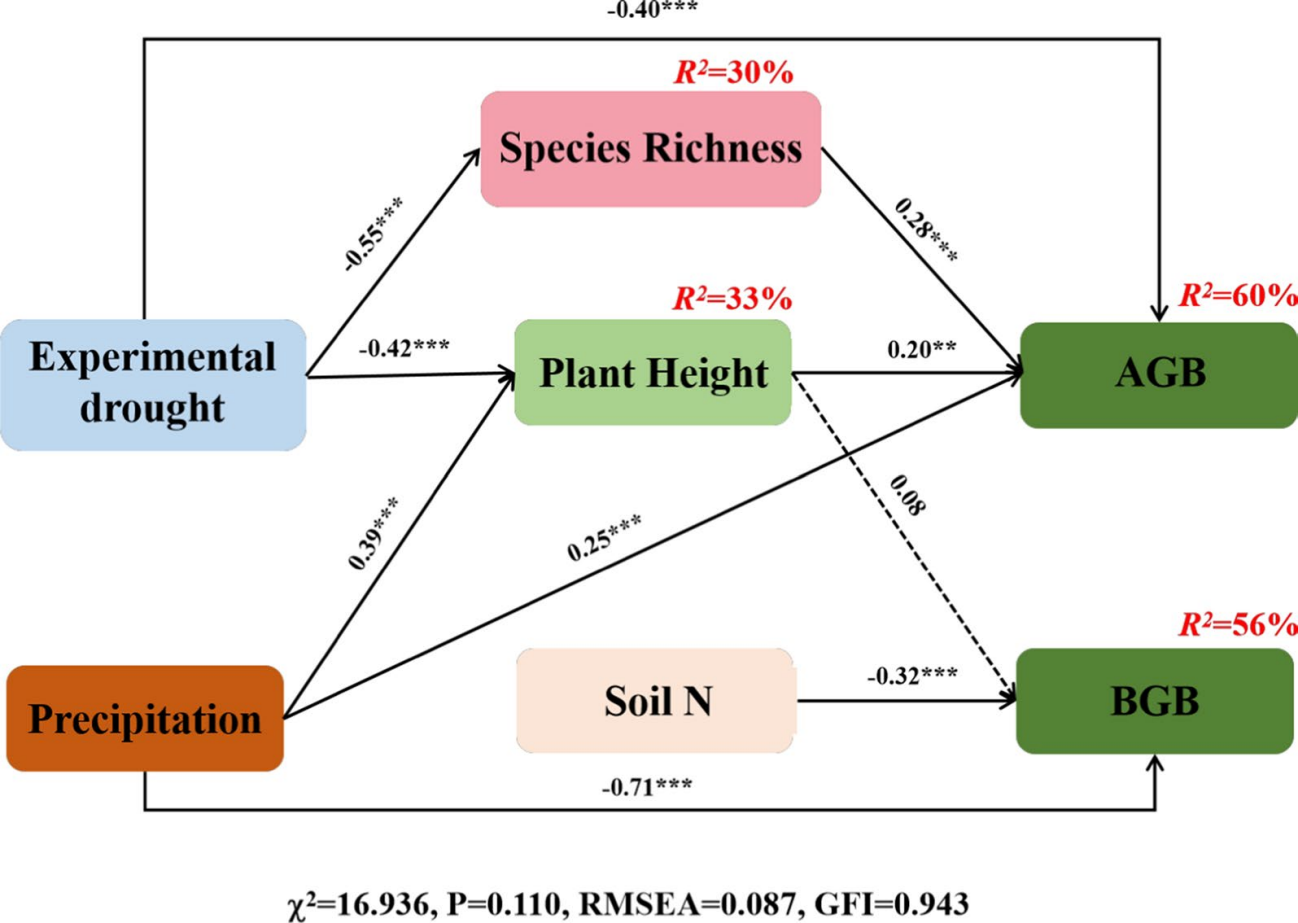
Structural equation modeling (SEM) depicting the effect paths of extreme drought treatments, precipitation in the early growing season (March to June), functional trait and soil properties on above- and belowground biomass. Square boxes indicate variables included in the model. Single headed arrows indicate paths. Numbers on path is the standardized regression weights. Total explained variance (R2) of biomass is on the right corner of boxes. Using the *, ** and*** to show the significance along the paths at the level of P < 0.05, P < 0.01and P < 0.001. Results of model fitting: ${\upchi }^{2}$=16.936, P = 0.110, RMSEA = 0.087, GFI = 0.943

As mentioned above, the precipitation in the early growing season (March to June) and experimental drought combining the two treatments were selected as exogenous variables in the structural equation model (SEM). The SEM was performed to quantify the direct vs indirect effects of how drought, precipitation in the early growing season (March to June), soil factors and CWM of plant traits on AGB or BGB. The model including the drought, precipitation, species richness, plant height and soil N was the best fit (χ^2^ = 16.936, P = 0.110; RMSEA = 0.087; GFI = 0.943) to explain 60% variance of AGB and 56% variance of BGB (Fig. 6).

The SEM models showed that increasing precipitation in the early growing season directly increased AGB and indirectly increased AGB through its positive impact on plant height (Table 1). The increasing plant height directly increased AGB and BGB (Table 1). The drought had a negative direct impact on AGB, also, the indirect impact of drought on AGB was through its negative impact on species richness and plant height (Table 1). The increasing species richness directly increased AGB (Table 1). Increasing soil N content and precipitation in the early growing season directly decreased BGB (Table 1).

**Table1:** The total, direct and indirect standardized effects on above- and belowground biomass from the structural equation model

Predictor	Pathways	Effect
Aboveground biomass		
Experimental drought	Direct	− 0.40
	Indirect	− 0.24
	Total	− 0.64
Precipitation	Direct	0.25
	Indirect	0.08
	Total	0.33
Height	Direct	0.20
	Indirect	NS
	Total	0.20
Species richness	Direct	0.28
	Indirect	NS
	Total	0.28
Belowground biomass		
Soil nitrogen content	Direct	− 0.32
	Indirect	NS
	Total	− 0.32
Precipitation	Direct	− 0.71
	Indirect	0.03
	Total	− 0.67
Height	Direct	0.09
	Indirect	NS
	Total	0.09

*NS* non-significant relationships

## Discussion

The response of ecosystems to changing precipitation is driven in part by species diversity and plant community functional traits. Thus, elucidating the variation of species diversity and CWM of traits under drought is critically important for improving predictions of ecosystem responses to changing precipitation. In semiarid grasslands of northern China, water is the limiting constraint to ecosystem development [45]. Here, we conducted an extreme drought experiment of four years to determine how desert steppe ecosystem modify plant community in response to the drought.

Our findings demonstrated that the species diversity was sensitive to experimental drought. We found that experimental drought (− 66% and − 60 Days), compared with the control, significantly reduced species richness in 2016–2018 (Fig. 1). Experimental drought (− 66% and − 60 Days) changed biodiversity that can be explained by species turnover/re-ordering caused by the cumulative effect of extreme drought (Additional file 1: Table S4). Experimental drought can modify species either through shifts in genotypic abundance and phenotypic plasticity by acting as an environment filter [46, 47]. On a temporal scale, there was no significant difference in species richness under experimental drought, which is contrary to other findings that suggested that plant species richness is more sensitive to drought in the arid ecosystem [2, 46]. One possible explanation for this difference could be the low soil moisture caused by extreme drought reduced the number of reproductive buds in many species [48, 49].

The relationship between functional traits of plants reflects the adaptation strategies of plants to the environment [46]. Plants usually adopt combinations of functional traits to adapt to changing environments [50]. In this study, we found that CWM of traits had no response to experimental drought but had significant response to natural drought (Fig. 1), which might be attributed to changes in species composition (Additional file 1: Table S4). We observed CWM of SLA and LNC increased, while CWM of plant height and LDMC decreased year by year. Previous studies respectively showed that plant height was significantly positively correlated with LDMC [50, 51], SLA was significantly negatively correlated with LDMC [52, 53], and SLA was significantly positively correlated with LNC [54, 55]. Our results are consistent with previous studies that showed that plants adapted to drought by changing leaf morphology and nutrient distribution [56, 57]. Not surprisingly, drought treatment of − 66% directly decreased soil water content except in 2017 (Fig. 3c, Additional file 1: Table S2). We also found that soil carbon and nitrogen content were not altered by experimental drought (− 66% and − 60 Days) (Fig. 3a, b, Additional file 1: Table S2). This can be explained by that drought can reduce plant nutrient input and increase soil nutrient loss, but also reduce soil nutrient loss by inhibiting soil organic matter decomposition [58, 59]. Plant nutrient contents usually reflect soil nutrient availability [60], however, we do not observe a match between plant nutrient concentrations and soil nutrient supply which also have been reported by other findings [61, 62]. This mismatch may be due to the lower soil moisture content, which results in limited nutrient flow and nutrient uptake by plants [63, 64].

Our results indicated that the aboveground biomass was significantly reduced by experimental drought treatment every year, which has been shown in several studies [65, 66]. However, the significant increase in belowground biomass due to experimental drought (-66% and -60 Days) occurred only in 2017. This difference from the optimal distribution theory may be due to the extreme drought alters in root distribution rather than the total amount of root biomass [67, 68]. Meanwhile, our findings demonstrated that AGB tended to decrease year by year and belowground biomass to increase, which in agreement with previous findings that have shown consecutive precipitation treatments can cause cumulative influence on ecosystem productivity [68, 69]. The positive relationships between species diversity and AGB are consistent with the results of the positive linear relationship common in species diversity—biomass relationship models [70, 71]. Our results showed that AGB was positively correlated with plant height, while BGB was negatively correlated with LDMC and plant height and positively correlated with SLA and LNC. These results support the other findings that some traits are good predictors of ecosystem function [72, 73]. The SEM results showed that CWM of plant height controlled by experimental drought and precipitation in the early growing season (March to June) exerted a direct effect on AGB. This is consistent with the CWM of traits determine the ecosystem function, which supports the mass ratio hypothesis [74, 75]. And it also proves that plant height is an important and comprehensive trait to reflect the ability of plants to adapt to changes in the environment [76]. Not surprisingly, drought and rainfall in March-June had direct impacts on AGB, confirming that in the previous findings [77, 78]. Our findings were consistent with others that precipitation and soil N had direct effects on belowground biomass [79]. These results suggest that precipitation in the early growing season has an important effect on plant biomass.

## Conclusion

This study showed that natural drought of early time in growing season can reduce the aboveground biomass and increased the belowground biomass, suggesting that the rainfall of early time in growing season plays an important role in maintaining ecosystem structure and function in desert steppe. Community-level plant height is an important predictor for AGB in desert steppe. Plant investment in the root system is a strategy for plants to adapt to soil nutrient reduction and drought of the early time in growing season, which provides deep insight into the mechanism of the above- and belowground biomass allocation of plants.

## Methods

### Experimental site

This study was conducted in the Urat Desert‐grassland Ecosystem Research Station (106° 58′ E, 41° 25′ N, 1,650 m above sea level) located in western Inner Mongolia, China. The region has a temperate continental monsoon climate, and the mean annual precipitation is 139.5 mm, about 70% occurring during the growing season [80]. The main soil type in the study area is brown calcium, and the dominant species in the desert steppe are *Stipa glareosa*, *Peganum harmala,* and *Allium polyrhizum* (Additional file 1: Table S4).

### Experimental treatments

The extreme drought experiment was established in 2014 and was conducted from 2015 to 2018. This experiment involved three treatments: (1) a control (ambient precipitation, without shelters), (2) a − 66% drought treatment (66% reduction from May 1 to August 31, with shelters), (3) and a − 60 Days drought treatment (100% reduction from June 1 to July 31, with shelters). There are eighteen 6 × 6 m plots in total, which are randomly distributed in location and organized into six blocks. Each plot was located at least 2 m from the nearest neighboring plot and established a 1-m external buffer to minimize the edge effects. To prevent hydrological exchange with the surrounding soil, a 1 m deep sheet of plastic flashing was established in each plot. The roofs consisted of strips of clear polycarbonate plastic was situated 2 m above the ground at the highest point, which allowed for the circulation of air and avoided microclimatic changes. Polycarbonate plastic has been confirmed to have minimal influence on photosynthetically active radiation [81].

### Sampling and analysis

During the peak of each growing season from 2015 to 2018, a quadrat (1 × 1 m) was set up in each experimental plot for vegetation investigation and sampling. Quadrat was marked to prevent subsequent resampling in the next year. We measured the maximum height of each species and recorded species richness (the number of plant species) in each quadrat. The density was defined as the number of plants per square meter. Besides, we harvested all aboveground biomass (AGB) by species in each quadrat. Finally, we estimated belowground biomass (BGB) using a root auger (8 cm diameter) to measure root mass at a depth of 0–20 cm. The roots samples were taken back to the laboratory and then were washed free of soil over a mesh sieve (mesh size of 0.25 mm). All above- and belowground biomasses were dried at 65 °C in an oven for 48 h and weighed in the lab.

We determined five key functional traits to reflect the plant morphology and growth investment [82, 83]: plant height, specific leaf area (SLA), leaf dry matter content (LDMC), leaf carbon content (LCC), and leaf nitrogen content (LNC). These traits were measured for the dominant species making up 90% of the total plant cover in each plot. The five traits on 10 individuals per species in each plot were obtained by using the standard methodologies [84]. We calculated community-weighted means (CWM) of single-trait by multiplying the trait value of each species by its relative biomass in the community [85]. CWM can reflect the characteristics of community functional traits [86]. In each plot, three soil samples (0–10 cm depth) were collected to determine soil water, and one mixed soil sample from three random replicates was collected to measure soil organic carbon and total nitrogen content. Leaf carbon and nitrogen content (%), as well as soil organic carbon and total nitrogen content (g Kg^−1^), were measured by using an Elemental Analyzer [36] (Costech ECS 4010, Italy) with a reduction temperature of 650 °C and a combustion temperature of 980 °C.

### Data analysis

We analyzed the response of each variable to extreme drought using separate repeated measures mixed model ANOVAs with year, treatment, and their interaction as fixed factor and block as a random factor (Additional file 1: Table S3). One-way ANOVA was conducted to assess the significant differences of species richness, Density, AGB, BGB, CWM of Height, CWM of SLA, CWM of LDMC, CWM of LCC, CWM of LNC, Soil Carbon, Soil Nitrogen, and Soil Water over to extreme drought among years. A level of P < 0.05 was considered significant. Data are presented as mean ± standard error throughout.

Then, the simple regression models with a standard 95% confidence range were used to assess whether CWM of traits and soil factors could explain AGB and BGB. We constructed a priori model (Additional file 1: Figure S2) based on the simple regression and the correlation coefficients of each variable (Additional file 1: Table S5). Drought treatment and the precipitation in the early time were treated as exogenous variables; species diversity, CWM of trait, and soil factors were considered as endogenous variables; AGB and BGB were regarded as the response variable. We assessed the best fitting model using a Chi-square test, root mean square error of approximation, and goodness-of-fit index [32], which was performed by AMOS 20.0 (Amos Development, Spring House, PA, USA). We eliminated the non-significant state variables and pathways by estimating regression weight estimates to simplify the initial model and finally obtained the final model containing the pathways that we failed to reject.

Data analysis and plotting were run with the SPSS16.0 and SigmaPlot12.0 for Windows statistics program, respectively. The simple regression models were performed using the *trendline* function in the *basic Trendline* package of R software (v4.0.0, R Core Team, 2020).

## Supplementary Information

**Additional file 1.** Additional figures and tables.

## Data Availability

The datasets used and/or analyzed during the current study are available from the corresponding author on reasonable request.

## Abbreviations

AGB: Aboveground biomass
BGB: Belowground biomass
CWM: Community-weighted means
SLA: Specific leaf area
LDMC: Leaf dry matter content
LCC: Leaf carbon content
LNC: Leaf nitrogen content
Soil N: Soil nitrogen content
