# Supplementary material for: Drought of early time in growing season decreases community aboveground biomass, but increases belowground biomass in a desert steppe
Source: BMC Ecol Evol. 2021 Jun 1;21:106. doi: 10.1186/s12862-021-01842-5 (PMC8170925; doi:10.1186/s12862-021-01842-5)
Supplement: Supplementary file 1 — Additional file 1. Additional figures and tables. [file 12862_2021_1842_MOESM1_ESM.docx]

**Additional file 1**

**
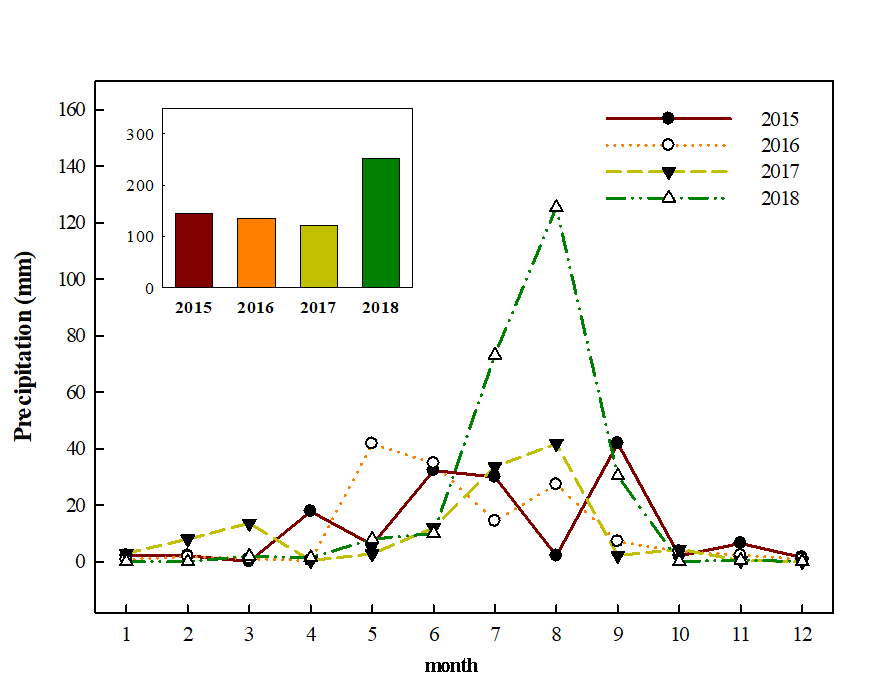
**

**Fig. S1** Monthly precipitation value of the research site from 2015 to 2018


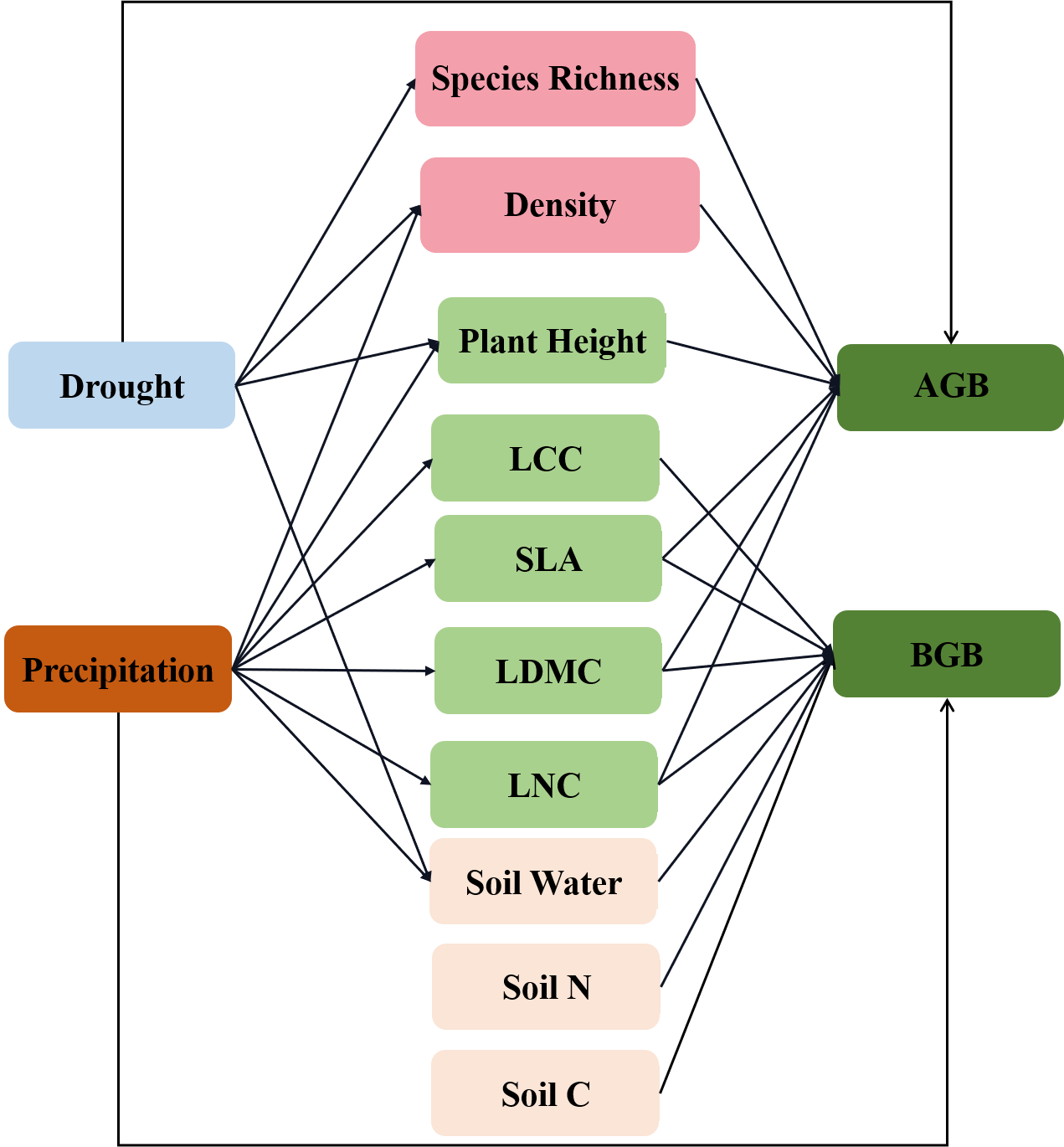


**Fig. S2** A‐priori structural equation model used in this study.


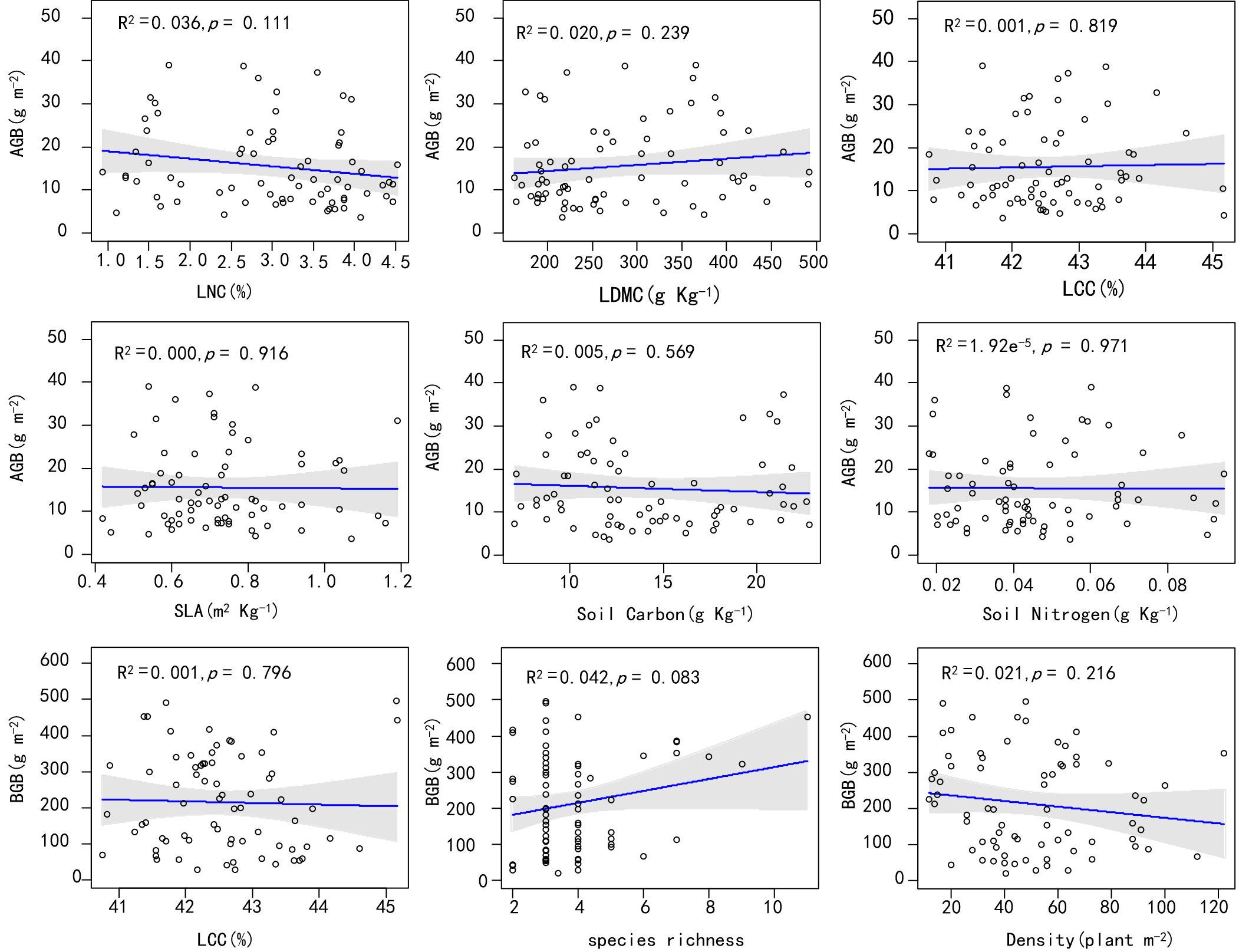


**Fig. S3** Relationships of community above-and belowground biomass with species diversity, community-weighted functional traits and soil factors across four years in the desert steppe. Only non-significant (p> 0.05) relationships were shown. Notes: SLA, specific leaf area; LDMC, leaf dry matter content; LNC, leaf nitrogen content; LCC, leaf carbon content.

**Table S1**. Correlation coefficients of above- and belowground biomass with [precipitation](C:/Users/DELL/AppData/Local/youdao/dict/Application/8.9.5.0/resultui/html/index.html#/javascript:;).

|  | [annual](C:/Users/DELL/AppData/Local/youdao/dict/Application/8.9.5.0/resultui/html/index.html#/javascript:;) [precipitation](C:/Users/DELL/AppData/Local/youdao/dict/Application/8.9.5.0/resultui/html/index.html#/javascript:;) | precipitation in growing season  (March to September) | precipitation in the early growing season (March to June) | AGB |
| --- | --- | --- | --- | --- |
| precipitation in growing season (March to September) | 0.999** |  |  |  |
| precipitation in the early growing season (March to June) | -0.543** | -0.523** |  |  |
| AGB | 0.025 | 0.032 | 0.302* |  |
| BGB | 0.480* | 0.480* | -0.663** | -0.154 |

*P < 0.05; **P < 0.01; ***P < 0.001.

**Table S2**. Comparison of plant biomass, species diversity, CWM of traits and soil factors difference between two drought treatments

|  | F | p |
| --- | --- | --- |
| AGB | 0.006 | 0.937 |
| BGB | 0.596 | 0.444 |
| Height | 2.200 | 0.145 |
| SLA | 0.157 | 0.693 |
| LDMC | 0.057 | 0.813 |
| LCC | 0.402 | 0.529 |
| LNC | 0.463 | 0.500 |
| Soil Water | 6.983 | 0.011 |
| Soil C | 0.008 | 0.929 |
| Soil N | 0.666 | 0.419 |
| Species Richness | 1.324 | 0.256 |
| Density | 0.222 | 0.640 |

**Table S3.** The separate repeated measures mixed model ANOVAs for the effects of extreme drought on community characteristics.

| Community characteristics | Drought | Year | Drought × Year |
| --- | --- | --- | --- |
| AGB | 35.662*** | 8.391*** | 2.455* |
| BGB | 1.123 | 46.609*** | 4.671*** |
| Height | 13.945*** | 11.126*** | 2.109^#^ |
| SLA | 0.555 | 55.798*** | 0.176 |
| LDMC | 0.08 | 38.237*** | 1.478 |
| LCC | 2.03 | 1.277 | 0.63 |
| LNC | 1.782 | 140.837*** | 0.559 |
| Soil water | 14.212*** | 26.248*** | 2.776* |
| Soil C | 0.017 | 4.929** | 0.874 |
| Soil N | 1.21 | 46.856*** | 1.073 |
| Species Richness | 31.325*** | 7.953*** | 9.748*** |
| Density | 3.581* | 11.29*** | 1.528 |

**#**, *****, ******, ******* and indicate statistically signiﬁcant levels at 0.1 < *p* < 0.05, *p* < 0.05, *p* < 0.01 and *p* < 0.001, respectively

**Table S4**. The effect of extreme drought on important values of plant species in community in desert steppe

|  | **2015** | | | | **2016** | | | | **2017** | | | | **2018** | | | |
| --- | --- | --- | --- | --- | --- | --- | --- | --- | --- | --- | --- | --- | --- | --- | --- | --- |
| species | | CONT | -66% | -60 Days | | CONT | -66% | -60 Days | | CONT | -66% | -60 Days | | CONT | -66% | -60 Days |
| *Stipa glareosa* | | 66.79±3.33 | 63.91±4.32 | 63.42±3.73 | | 33.25±4.15 | 24.14±3.94 | 32.95±2.30 | | 22.14±2.69 | 28.18±5.97 | 20.84±2.72 | | 12.06±1.82 | 9.73±0.6 | 38.43±2.83 |
| *Peganum harmala* | | 16.97±2.22 | 24.81±5.57 | 23.91±4.34 | | 30.47±4.12 | 36.16±5.04 | 33.39±4.74 | | 26.06±3.21 | 33.95±7.81 | 37.53±3.41 | | 16.86±1.87 | 42.96±3.66 | 38.33±3.68 |
| *Allium polyrhizum* | | 7.84±1.39 | 8.27 |  | | 20.38±2.79 | 27.12±6.60 | 32.31±3.45 | | 45.86±2.92 | 37.87±7.35 | 37.12±4.92 | | 51.22±2.38 | 42.29±4.01 | 45.46±5.62 |
| *Ajania fruticulosa* | | 13.39±0.23 |  |  | | 22.93±16.15 | 14.57 |  | | 7.60 |  |  | | 1.51±0.18 |  |  |
| *Asparagus gobicus* | | 4.23 | 10.51±2.12 | 15.32 | | 8.62±1.43 | 18.43±5.38 | 8.08 | |  |  | 9.52 | | 5.64±1.04 | 7.24 | 8.7±1.07 |
| *Artemisia frigida* | | 7.21±0.91 | 5.60 | 13.99 | |  |  |  | | 14.01 |  |  | | 2.42±0.35 |  |  |
| *Allium mongolicum* | |  |  |  | |  |  |  | |  |  |  | | 7.15±1.39 |  |  |

Note: Variables are shown as mean ± SE (n = 6); CONT, control; -66%, reduce 66% in rainfall from May to August; -60 Days, reduce 100% in rainfall from June to July. Species importance value = (relative height + relative cover + relative biomass) / 3

**Table S5**. Correlation coefficients of above- and belowground biomass with species diversity, community-weighted trait and soil properties.

|  | Drought | Precipitation | AGB | BGB | Height | SLA | LDMC | LCC | LNC | Soil Water | Soil C | Soil N | Species Richness |
| --- | --- | --- | --- | --- | --- | --- | --- | --- | --- | --- | --- | --- | --- |
| Precipitation | 0 |  |  |  |  |  |  |  |  |  |  |  |  |
| AGB | -0.645** | 0.302* |  |  |  |  |  |  |  |  |  |  |  |
| BGB | 0.028 | -0.663** | -0.154 |  |  |  |  |  |  |  |  |  |  |
| Height | -0.421** | 0.395** | 0.550** | -0.267* |  |  |  |  |  |  |  |  |  |
| SLA | -0.051 | -0.658** | -0.068 | 0.649** | -0.394** |  |  |  |  |  |  |  |  |
| LDMC | 0.005 | 0.449** | 0.141 | -0.558** | 0.353** | -0.793** |  |  |  |  |  |  |  |
| LCC | -0.234* | 0.046 | 0.022 | -0.139 | 0.081 | -0.184 | 0.309** |  |  |  |  |  |  |
| LNC | 0.013 | -0.576** | -0.190 | 0.696** | -0.414** | 0.753** | -0.851** | -0.285* |  |  |  |  |  |
| Soil Water | -0.282* | -0.197 | 0.345** | 0.471** | -0.116 | 0.557** | -0.334** | -0.070 | 0.397** |  |  |  |  |
| Soil C | -0.018 | -0.197 | -0.013 | 0.376** | 0.059 | 0.172 | -0.273* | -0.120 | 0.340** | 0.247* |  |  |  |
| Soil N | 0.039 | -0.092 | -0.004 | -0.244* | 0.204 | -0.354** | 0.564** | 0.129 | -0.566** | -0.259* | 0.008 |  |  |
| Species Richness | -0.548** | -0.111 | 0.530** | 0.206 | 0.271* | 0.290* | -0.224 | -0.040 | 0.157 | 0.374** | 0.115 | -0.12 |  |
| Density | -0.249* | 0.508** | 0.459** | -0.148 | 0.536** | -0.312** | 0.124 | -0.007 | -0.168 | -0.004 | 0.085 | -0.115 | 0.199 |

Drought, extreme treatments (CONT; -66%; -60Days); Precipitation, the precipitation in the early growing season (March to June); AGB, aboveground plant biomass; BGB, belowground root biomass; SLA, specific leaf area; LDMC, leaf dry matter content; LNC, leaf N content; LCC, leaf C content; *P < 0.05; **P < 0.01; ***P < 0.001
